# Chromosome-scale genome assembly of Japanese pear (*Pyrus pyrifolia*) variety ‘Nijisseiki’

**DOI:** 10.1093/dnares/dsab001

**Published:** 2021-02-26

**Authors:** Kenta Shirasawa, Akihiro Itai, Sachiko Isobe

**Affiliations:** 1 Department of Frontier Research and Development, Kazusa DNA Research Institute, Chiba, Japan; 2 Graduate School of Life and Environmental Sciences, Kyoto Prefectural University, Kyoto, Japan

**Keywords:** chromosome-scale genome assembly, genome-wide duplication, Japanese pear, long-read sequencing technology

## Abstract

We analyzed the genome sequence of a Japanese pear (*Pyrus pyrifolia*) to facilitate its genetics and genomics as well as breeding programs, in which a variety 'Nijisseiki' with superior flesh texture has been used as a parent for most Japanese pear cultivars. De novo assembly of long sequence reads covered 136× of the Japanese pear genome and generated 503.9 Mb contigs consisting of 114 sequences with an N50 value of 7.6 Mb. Contigs were assigned to Japanese pear genetic maps to establish 17 chromosome-scale sequences. In total, 44,876 high-confidence protein-encoding genes were predicted, 84.3% of which were supported by predicted genes and transcriptome data from Japanese pear relatives. As expected, evidence of genome-wide duplication was observed, consistent with related species. This is the first chromosome-scale genome sequence analysis reported for Japanese pear, and this resource will support breeding programs and provide new insights into the physiology and evolutionary history of Japanese pear.

## 1. Introduction

Pear (*Pyrus* spp.) is a genus of the Malinae subtribe of the Rosaceae that includes European pear (*P. communis*), Chinese white pear (*P.* × *bretschneideri*), Japanese pear (*P. pyrifolia*), and apple (*Malus* × *domestica*). The Japanese pear variety ‘Nijisseiki’ (a Japanese term referring to the 20th century) was discovered in Matsudo (Chiba, Japan) in 1888.^1^ Due to its favourable flesh texture, ‘Nijisseiki’ was the leading Japanese pear variety in Japan from the 1940s to the 1980s, and ‘Nijisseiki’ was widely used as a breeding parent for the development of Japanese pear cultivars in Japan.^1^

Genome information can enhance breeding programs^2^ by facilitating understanding of the genetic backgrounds of the breeding pedigrees^3^ and by owing to the advancements on physiology and evolutionary studies. In the Malinae, genome sequence data are publicly available for species such as apple,^4^ European pear,^5^ and Chinese white pear,^6^ as well as for some pear wild relatives (*P. betulifolia* and (*P. ussuriensis* × *communis*) × spp.) that are used for root stocks.^7^^,^^8^ Genome analysis of the Malinae is complex due to ancestral genome-wide duplication^9^ and high heterozygosity in their genomes resulting from allogamy and self-incompatibility. In apple^4^ and European pear,^5^ doubled-haploid lines were developed to reduce the genome complexities to simplify the de novo genome sequencing analysis. However, neither doubled-haploid lines nor genome sequence data are available for Japanese pear, despite the publication of transcriptome data^10^ and genetic maps.^11^^,^^12^

Long-read sequencing, also known as single-molecule real-time sequencing (PacBio, Menlo Park, CA, USA) and nanopore sequencing (Oxford Nanopore Technologies, Oxford, UK), has several advantages over short-read technologies based on a sequencing by synthesis method (Illumina, San Diego, CA, USA and MGI Tech, Shenzhen, China).^13^ Long reads are capable to span repetitive sequences in genomes, extending sequence contiguity and facilitating assembly. Furthermore, long reads allow haplotype phases of highly heterozygous genome sequences to be determined. In this study, the single-molecule real-time long-read sequencing was used to produce a highly contiguous genome sequence assembly of the Japanese pear variety ‘Nijisseiki’. This Japanese pear genome will enhance our understanding of genetics, genomics, and breeding in Japanese pear.

## 2. Materials and methods

### 2.1. Plant materials and DNA extraction

A single tree of Japanese pear (*P. pyrifolia*), variety ‘Nijisseiki’, which is planted at the orchard of Kyoto Prefectural University (Kyoto, Japan), was used for genome sequencing analysis. Genome DNA was extracted from the young leaves by a modified sodium dodecyl sulphate method.^14^

### 2.2. Estimation of the genome size of Japanese pear

A short-read sequence library was prepared using a PCR-free Swift 2S Turbo Flexible DNA Library Kit (Swift Sciences, Ann Arbor, MI, USA) and converted into a DNA nanoball sequencing library with an MGI Easy Universal Library Conversion Kit (MGI Tech). The library was sequenced on a DNBSEQ G400RS (MGI Tech) instrument in paired-end, 101-bp mode. The obtained reads were used to estimate the genome size with Jellyfish after removing low-quality bases (<10 quality value) with PRINSEQ, adaptor sequences (AGATCGGAAGAGC) with fastx_clipper in FASTX-Toolkit, and reads from organelle genomes (GenBank accession numbers: AP012207 and KY563267)^15^^,^^16^ by read mapping with Bowtie2 on the reference sequences.

### 2.3. Chromosome-scale genome assembly

The software tools used for the data analyses are shown in Supplementary Table S1. A long-read sequence library was constructed using an SMRTbell Express Template Prep Kit 2.0 and sequenced on SMRT cells (1 M v3 LR) in a PacBio Sequel system (PacBio). The obtained reads (≥15 kb) were assembled with Falcon, and the two haplotype sequences, primary contigs (from one haploid genome) and haplotigs (from the other haploid), of the high heterozygous diploid genome were resolved with Falcon-unzip. Potential sequence errors in the assembled sequences were corrected with ARROW using the PacBio reads. Haplotype duplications in the primary contigs were removed with Purge_Dups. Sequences from the organelle genomes, which were identified by a sequence similarity search of reported plastid (AP012207)^16^ and mitochondrial (KY563267)^15^ genome sequences from Japanese pear using minimap2, were also deleted. The final assembly was designated as PPY_r1.0. The primary contigs of PPY_r1.0 were assigned to genetic maps of Japanese pear^11^^,^^12^ with ALLMAPS, in which marker sequences were aligned on the contigs with BLAST. The resultant chromosome-scale pseudomolecule sequences were named PPY_r1.0.pmol. The haplotig sequences were aligned onto PPY_r1.0.pmol with minimap2. Completeness evaluation of the assembly was performed with Benchmarking Universal Single-Copy Orthologs (BUSCO, embryophyta odb9), in which copy number of the core gene set present in single copy in most Embryophyta is assessed. The chromosome-scale pseudomolecule sequences (PPY_r1.0.pmol) were compared with those of apple (GDDH13, v1.1),^4^ European pear (Bartlett DH, v2.0),^5^ and Chinese white pear (Dangshan Suli, v1.1)^6^ with D-GENIES. Genome-wide duplications were detected with MCScanX, with threshold values of ≥85% sequence identity and ≤1e-100 E-values, and visualized with VGSC.

### 2.4. Gene prediction and repetitive sequence analysis

Protein-encoding genes were predicted by a MAKER pipeline. Two presets of Augustus and SNAP for Arabidopsis were used as training data for *ab initio* prediction. In addition, peptide sequences of apple (GDDH13, v1.1),^4^ European pear (Bartlett DH, v2.0),^5^ and Chinese white pear (Dangshan Suli, v1.1)^6^ registered in the Genome Database for Rosaceae (GDR),^17^ as well as transcriptome data for Japanese pear (Ppyrifolia_protein_v1.0),^10^ were also employed in the prediction based on the protein homology evidence. Short gene sequences of <300 bases, as well as genes predicted with an annotation edit distance (AED) of >0.5 which threshold is proposed as good annotations in the MAKER protocol, were removed to facilitate selection of high-confidence genes. The genome positions of the high-confidence genes were compared with those of the peptide sequences from apple, European pear, Chinese white pear, and Japanese pear, and were aligned to PPY_r1.0.pmol in the MAKER pipeline. Functional annotation of the predicted genes was performed with Hayai-Annotation Plants.

Repetitive sequences in the PPY_r1.0.pmol assembly were identified with RepeatMasker, using repeat sequences registered in Repbase and a de novo repeat library built with RepeatModeler. The repeat elements were classified into nine types: short interspersed nuclear elements (SINEs), long interspersed nuclear elements (LINEs), long terminal repeat (LTR) elements, DNA elements, Small RNA, satellites, simple repeats, low complexity repeats, and unclassified, in accordance with RepeatMasker.

## 3. Results and data description

### 3.1. Assembly of the genome of a Japanese pear, ‘Nijisseiki’

**Figure 1:**
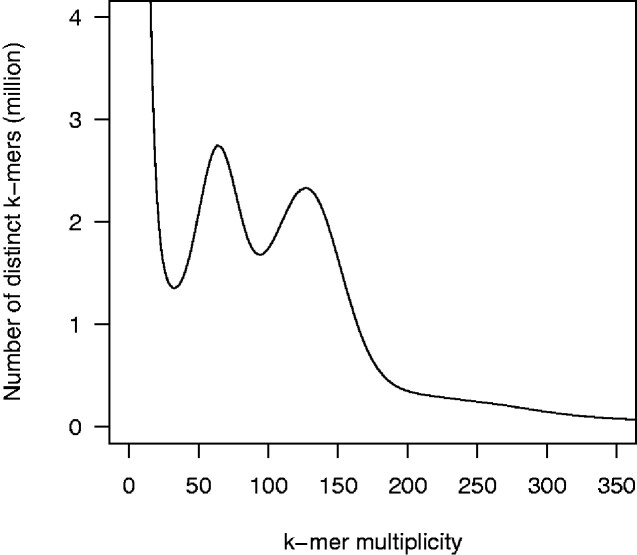
Genome size estimation for Japanese pear ‘Nijisseiki’ with the distribution of the number of distinct *k*-mers (*k* = 17) with the given multiplicity values.

In total, 76.9 Gb short-read data were used for estimation of the genome size of Japanese pear. *K*-mer distribution analysis indicated that the ‘Nijisseiki’ genome was highly heterozygous, and that the estimated haploid genome size was 537.1 Mb (Fig. 1), which almost agreed with the value measured by flow cytometry, 503.50 ± 31.83 Mb.^18^

Long-read sequencing analysis with four SMRT cells generated 85.8 Gb data (4.5 million reads with an N50 of 25.5 kb). Of these, reads ≥15 kb in length (73.3 Gb, 136.4× genome coverage) were selected and assembled into 750.0 Mb contigs (495 sequences with an N50 of 5.9 Mb). The contig sequences were resolved into primary contigs and haplotigs, and polished to correct potential errors. The sizes of the resultant sequences were 745.8 Mb for the primary contigs and 113.3 Mb for the haplotigs, which represented genome sequences of one haploid genome sequence and another haploid of highly heterozygous regions in the diploid genome, respectively. As this primary contig size was longer than the estimated haploid size, we doubted the existence of duplicate sequences. Hence, 257 haplotype duplications in the primary contigs (241.6 Mb) identified by a read depth distribution pattern of Purge_Dups (Supplementary Figure S1) were excluded. In addition, 10 sequences (283.5 kb), >99% of which lengths were aligned onto the previously reported organelle genomes with minimap2, were removed. The final assembly, named PPY_r1.0, consisted of 503.9 Mb primary contigs (including 114 sequences with an N50 length of 7.6 Mb) and 353.4 Mb haplotigs (including 822 sequences with an N50 length of 1.6 Mb) (Table 1). A BUSCO analysis of the primary contigs indicated that 58.3% and 39.7% of sequences were single-copy and duplicated complete BUSCOs, respectively (Table 2), supporting the genome-wide duplication state in the Japanese pear genome.

**Table 1:** Statistics of the primary contig sequences of Japanese pear ‘Nijisseiki’

	PPY_r1.0 (primary contigs)	PPY_r1.0. haplotigs (haplotig contigs)	PPY_r1.0.pmol (pseudomolecule sequences)
Total contig size (bp)	503,888,133	353,451,501	481,431,185
Number of sequences	114	822	17
Sequence N50 length (bp)	7,676,629	1,608,651	31,638,135
Longest sequence size (bp)	28,225,311	7,932,431	39,634,718
Gap size (bp)	0	0	7,200
#Predicted genes	44,876	Not available	43,065

**Table 2:** Completeness evaluation of genome assembly and predicted genes

	Genome (PPY_r1.0)	Predicted genes
Complete BUSCOs	98.00%	98.40%
Single-copy BUSCOs	58.30%	58.60%
Duplicated BUSCOs	39.70%	39.80%
Fragmented BUSCOs	0.50%	0.90%
Missing BUSCOs	1.50%	0.70%

### 3.2. Chromosome-scale assembly construction and comparative genomics

Two previously established genetic maps for Japanese pear^11^^,^^12^ were used to establish pseudomolecule sequences. Totals of 609 and 2,388 SNP marker sequences were collected from the genetic maps of Li et al.^11^ and Terakami et al.,^12^ respectively, and used to identify 587 and 2,306 marker positions on the primary contigs, respectively. Thus, 89 contig sequences (481.4 Mb in total) were assigned to the 17 linkage groups with the marker positions as anchors and connected with 100 Ns to create pseudomolecule sequences (Tables 1 and 3). The remaining 25 contigs (22.4 Mb) were not assigned to any linkage groups. The haplotig sequences were aligned on the pseudomolecule sequences and covered 332.5 Mb in length. In total, 1,719,266 allelic sequence variants were detected between the two haplotypes.

**Table 3:** Statistics of the Japanese pear ‘Nijisseiki’ pseudomolecule sequences, PPY_r1.0.pmol

Chromosome	#Contigs	%	Total contig size (bp)	%	#Genes	%
Chr01	3	2.6	23,195,497	4.6	2,121	4.7
Chr02	7	6.1	31,777,058	6.3	2,828	6.3
Chr03	6	5.3	31,637,635	6.3	2,765	6.2
Chr04	5	4.4	19,501,643	3.9	1,625	3.6
Chr05	3	2.6	35,298,035	7.0	3,320	7.4
Chr06	6	5.3	22,071,348	4.4	2,016	4.5
Chr07	8	7.0	36,191,613	7.2	3,009	6.7
Chr08	6	5.3	26,500,184	5.3	2,508	5.6
Chr09	3	2.6	24,999,441	5.0	2,399	5.3
Chr10	2	1.8	21,149,732	4.2	2,159	4.8
Chr11	7	6.1	35,209,706	7.0	2,788	6.2
Chr12	5	4.4	24,643,494	4.9	2,227	5.0
Chr13	6	5.3	29,570,600	5.9	2,662	5.9
Chr14	8	7.0	22,573,349	4.5	2,119	4.7
Chr15	4	3.5	39,634,418	7.9	3,697	8.2
Chr16	5	4.4	25,480,041	5.1	2,187	4.9
Chr17	5	4.4	31,990,191	6.3	2,635	5.9
**Subtotal (Chr01-Chr17)**	**89**	**78.1**	**481,423,985**	**95.5**	**43,065**	**96.0**
Unassigned contigs	25	21.9	22,747,703	4.5	1,811	4.0
**Total**	**114**	**100.0**	**504,171,688**	**100.0**	**44,876**	**100.0**

**Figure 2:**
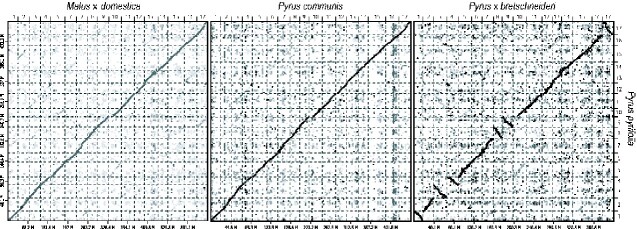
Comparative analysis of sequence and structure similarities of the Japanese pear genome. Genome sequence and structure similarities of Japanese pear (*P. pyrifolia*) with apple (*M.* × *domestica*), European pear (*P. communis*), and Chinese white pear (P. × bretschneideri).

**Figure 3:**
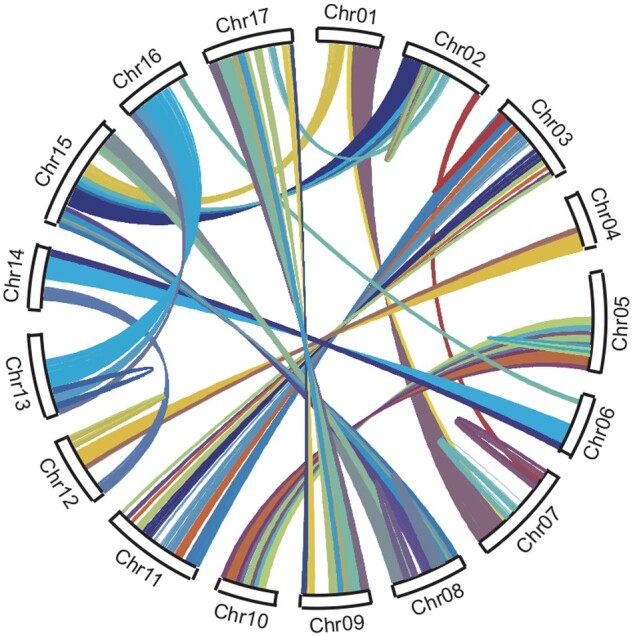
Genome-wide duplication of the Japanese pear genome. Genome regions showing sequence similarities are connected with lines.

The structure of the pseudomolecule sequences was compared with those of apple, European pear, and Chinese white pear to reveal one-to-one synteny relationships at chromosome level between the species (Fig. 2). Furthermore, possible homologous chromosome pairs derived from genome-wide duplication and intra-chromosomal duplications were detected (Fig. 3).

### 3.3. Gene prediction and repetitive sequence analysis

Initial predictions suggested 85,013 likely genes in the genome assembly. This gene number was resolved to 44,876 (Table 1) after removal of 29,607 low-confidence genes with an annotation edit distance of >0.5 (Supplementary Figure S2), 10,527 short genes of <300 bases in length, and three genes in redundant positions. A BUSCO analysis of the 44,876 remaining genes indicated that 98.4% of the sequences were complete BUSCOs (Table 2). The 44,876 sequences (56.0 Mb in total length) were therefore concluded to be high-confidence Japanese pear genes.

**Figure 4:**
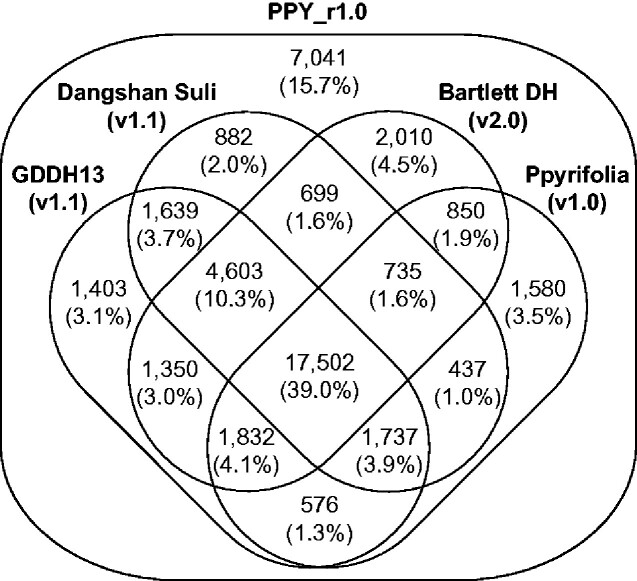
Number of genes supported by members of the Rosaceae. The predicted genes in the genomes of the Japanese pear (PPY_r1.0), apple (GDDH13, v1.1),^4^ Chinese white pear (Dangshan Suli, v1.1),^6^ European pear (Bartlett DH, v2.0),^5^ and the transcriptome of Japanese pear (Ppyrifolia_protein_v1.0).^10^

The genome positions of 37,835 (84.3%) of the 44,876 genes were covered with those of transcripts of Japanese pear and/or peptide sequences predicted from the genomes of apple, European pear, and Chinese white pear (Fig. 4). Functional gene annotation analysis revealed that 6,610 (14.7%), 11,664 (26.0%), and 8,168 (18.2%) sequences were assigned to Gene Ontology slim terms in the biological process, cellular component, and molecular function categories, respectively, and 1,967 genes had enzyme commission numbers (Supplementary Table S2).

Repetitive sequences occupied a total of 280.3 Mb (55.6%) of the PPY_r1.0.pmol (503.8 Mb) assembly. The repeats consisted of nine major types in varying proportions (Table 4). The dominant repeat types in the pseudomolecule sequences were LTR retroelements (172.9 Mb) followed by DNA transposons (59.4 Mb). Repeat sequences that were unavailable in public databases totalled 25.0 Mb.

**Table 4:** Repetitive sequences in the Japanese pear ‘Nijisseiki’ sequences, PPY_r1.0

Repeat type	Number of elements	Length occupied (bp)	%^a^
SINEs	27,012	3,844,706	0.80
LINEs	21,048	7,222,875	1.40
LTR elements	162,422	172,911,968	34.30
DNA transposons	246,048	59,478,780	11.80
Unclassified	131,375	25,065,317	5.00
Small RNA	27,106	4,153,084	0.80
Satellites	648	109,271	0.00
Simple repeats	121,341	4,868,197	1.00
Low complexity	20,350	974,726	0.20

a Percentage of sequence length of PPY_r1.0 (503,888,133 bp).

## 4. Conclusion and future perspectives

Here, we present the first reported genome sequence analysis of Japanese pear, complementing the existing available genome sequences of other members of the *Pyrus* genus.^5–8^ Long-sequencing technology allowed the assembly of 114 contig sequences (N50 of 7.6 Mb) spanning 503.9 Mb, 89.6% of the estimated size of the genome (Table 1). Subsequently, 481.4 Mb (95.5% of the assembled sequences) were successfully assigned to the Japanese pear chromosomes to establish pseudomolecule sequences, PPY_r1.0.pmol. This pseudomolecule assembly is the longest among the reported *Pyrus* spp.,^5–8^ and PPY_r1.0.pmol can thus act as a new standard for *Pyrus* genomics. Genome structures and genome-wide duplication events were conserved among pears and apples (Figs 2 and 3) despite their divergent genome sizes.^2^^,^^4–8^ This Japanese pear genome assembly contributes to our understanding of the evolutionary history of pear and other Malinae species.

The ‘Nijisseiki’ variety of Japanese pear was used for the genome analysis. Although the parents of the ‘Nijisseiki’ variety are unknown, its favourable flesh texture^1^ led to its use as a popular breeding parent for Japanese pear cultivars. The genome data generated in this study will facilitate the discovery of the likely parents of the ‘Nijisseiki’ variety and will allow the ‘Nijisseiki’ haplotypes to be traced in progeny cultivars. As the superior flesh texture was unique to ‘Nijisseiki’ at the point of its discovery, and this texture was passed to progeny cultivars,^1^ it should be possible to discover the genomic region responsible for the texture trait through pedigree analysis. Moreover, discovery of the ancestor lines of ‘Nijisseiki’ would be helpful to understand the origin of the superior trait even though the parents may be already lost. This approach was used successfully to find associations between haplotypes and phenotypes for five traits in apple cultivars bred in Japan, for which the popular ‘Fuji’ variety was the founder.^3^

The Japanese pear genome will provide new insights into the physiology and evolutionary history of Malinae and will facilitate Japanese pear breeding programs. Further analysis will perhaps contribute to identify the relevant genetic mechanisms for favourable flesh texture of ‘Nijisseiki’ and its progeny cultivars.

## Supplementary data

Supplementary data are available at DNARES online.

## Supplementary Material

dsab001_Supplementary_Data
