# Supplementary material for: Chromosome-scale genome assembly of Japanese pear (Pyrus pyrifolia) variety ‘Nijisseiki’
Source: DNA Res. 2021 Feb 26;28(2):dsab001. doi: 10.1093/dnares/dsab001 (PMC8092371; doi:10.1093/dnares/dsab001)
Supplement: dsab001_Supplementary_Data [file dsab001_supplementary_data.zip › 20Cgenome-SupplementaryFiguresR1.pdf]

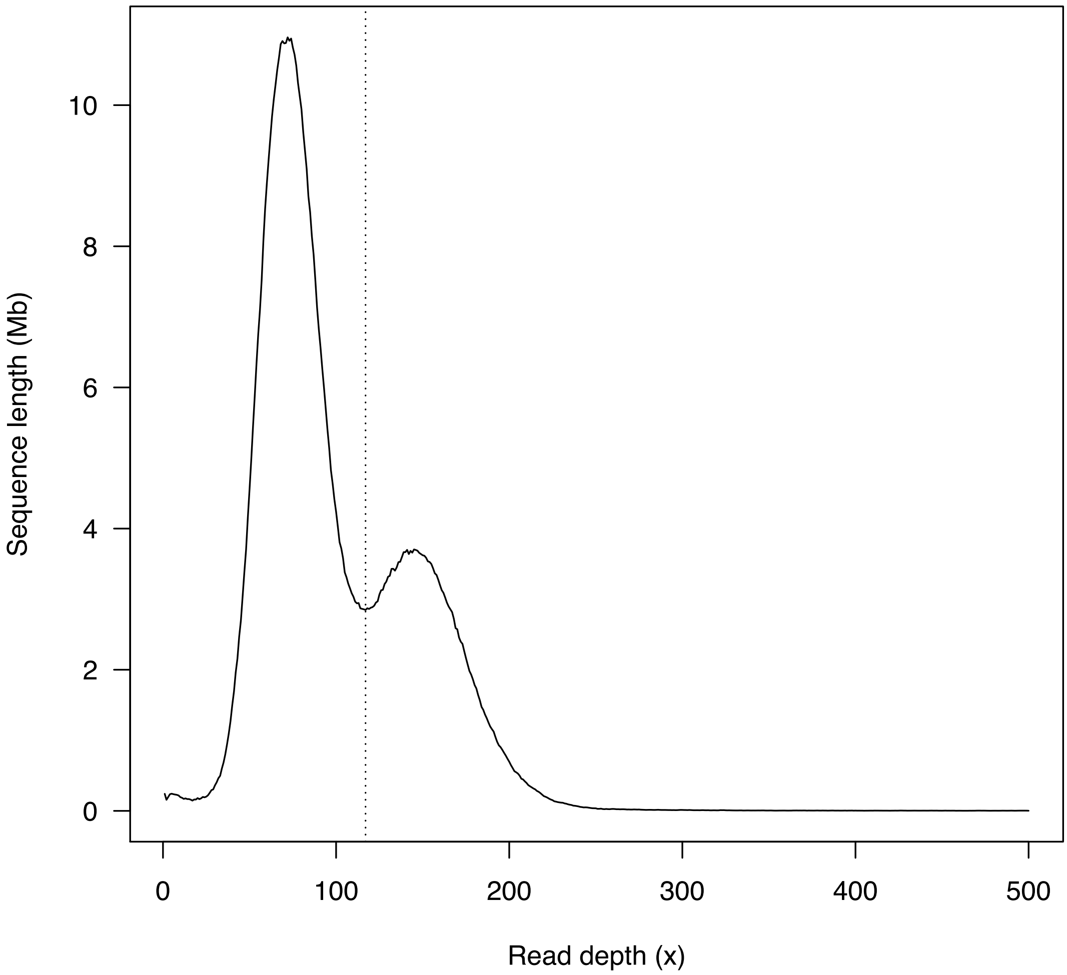

Supplementary Figure S1 Distribution of read depth on the primary genome assemblies. A cutoff value at read depth of >117 to delete duplicated sequences are indicated by a dotted line.

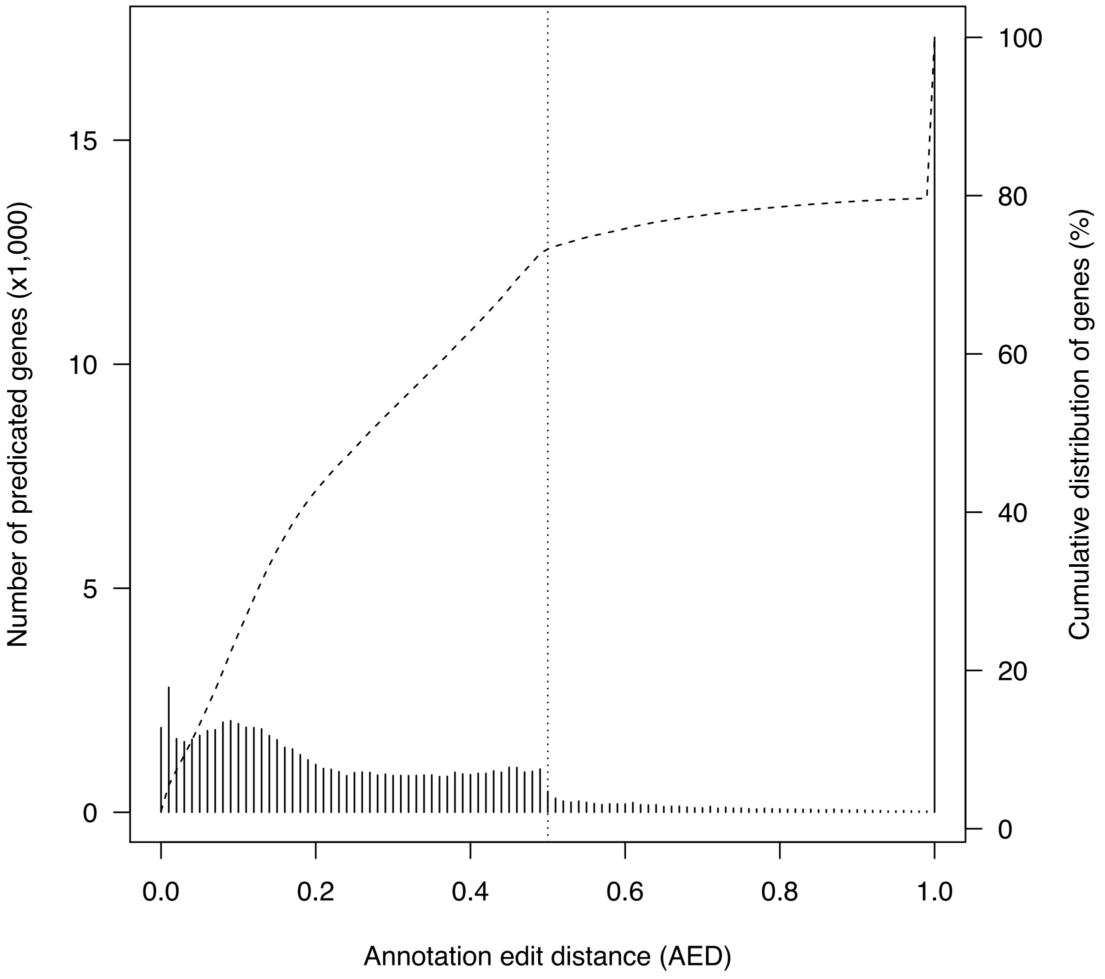

Supplementary Figure S2 Annotation edit distance (AED) calculated for all of genes predicted with a MAKER pipeline.

Bars and a dashed line indicate number of predicted genes (left y-axis) and cumulative distribution functions of the genes (right y-axis), respectively. A vertical dotted line is a threshold AED score of 0.5.
